# Two novel species of sooty mould fungi (Capnodiales, Dothideomycetes) from China

**DOI:** 10.3897/mycokeys.129.182290

**Published:** 2026-02-19

**Authors:** Jipeng Sun, Entaj Tarafder, Tao Chen, Sinang Hongsanan, Shengxi Chen, Chada Norphanphoun, Samantha C. Karunarathna, Xiangyu Zeng

**Affiliations:** 1 Department of Plant Pathology, Agricultural College, Guizhou University, Guiyang 550025, China Shenzhen University Shenzhen China; 2 Guizhou Key Laboratory of Agricultural Microbiology, Guiyang 550025, China Qujing Normal University Qujing China; 3 Institute of Edible Mushrooms, Guizhou University, Guiyang 550025, China Agricultural College, Guizhou University Guiyang China; 4 Center for Yunnan Plateau Biological Resources Protection and Utilization & Yunnan International Joint Laboratory of Fungal Sustainable Utilization in South and Southeast Asia, College of Biology and Food Engineering, Qujing Normal University, Qujing 655099, China Institute of Edible Mushrooms, Guizhou University Guiyang China; 5 Public Health and Health Sciences College, Guizhou Medical University, Guiyang 551113, China Guizhou Medical University Guiyang China; 6 Shenzhen Key Laboratory of Microbial Genetic Engineering, College of Life Science and Oceanography, Shenzhen University, Shenzhen 518060, China Guizhou Key Laboratory of Agricultural Microbiology Guiyang China

**Keywords:** Capnodiaceae, fungal diversity, new species, phylogenetic analysis, taxonomy

## Abstract

*Conidiocarpus* and *Polychaeton* are genera of sooty mould fungi (Capnodiales, Dothideomycetes) that grow epiphytically on the leaf surfaces of living plants and typically depend on honeydew excreted by sap-sucking insects as their primary nutrient source. Heavy colonization by these fungi may impair host photosynthesis, reduce plant vigor, and decrease the aesthetic or economic value of affected plants. In this study, two novel species, *Conidiocarpus nanshanense* and *Polychaeton cengongense*, were collected from the leaves of *Schefflera macrostachya* (large-spiked schefflera) and *Dalbergia assamica* (South China rosewood) in Guizhou and Guangdong Provinces, respectively. Species delimitation was based on detailed morphological characteristics and phylogenetic analyses of multiple loci, including ITS, LSU, *tef*1-α, and *rpb*2. Morphological descriptions and illustrations are provided, together with phylogenetic analyses inferred using maximum likelihood (ML) and Bayesian inference (BI). The results expand the known taxonomic diversity of sooty mould fungi in China and provide important morphological and molecular data that support species delimitation and classification within Capnodiales.

## Introduction

Capnodiaceae (Capnodiales, Dothideomycetes), a family of epiphytic sooty mould fungi, was first established by Höhnel (1910), with *Capnodium* designated as the type genus (Kirk et al. 2008; Lumbsch and Huhndorf 2011). The taxonomic status of the family was formally validated by Theissen (1915). Currently, this family comprises 11 genera and approximately 150 species (Lu et al. 2022; Wijayawardene et al. 2022; Yang et al. 2025). Among these genera, *Scoriadopsis*, *Limaciniaseta*, *Kosmimatomyces*, and *Hyphocapnodia* are monotypic and lack molecular data (Lu et al. 2022; Wijayawardene et al. 2022), with only morphological descriptions available. Hughes (1976) published the first monograph on sooty mould fungi, systematically summarizing the entire group. Subsequent researchers have further refined the morphological descriptions and supplemented molecular data for species in this family based on this work (Crous et al. 2009; Chomnunti et al. 2011; Abdollahzadeh et al. 2020).

Capnodiaceae is the representative family of sooty mould fungi (Crous et al. 2007; Abdollahzadeh et al. 2020). Its key characteristics include septate, dark-brown hyphae that form a thin hyphal network on the host surface; bitunicate asci; and asexual morphs producing slender conidiomata with short or long, narrow necks (Chomnunti et al. 2011). Distinct elliptical swellings are present near the base, middle, or apex of the conidiomata, and transparent conidia are produced inside these swollen regions (Chomnunti et al. 2011; Abdollahzadeh et al. 2020). Sooty mould refers to a group of epiphytic fungi that obtain nutrients from honeydew secreted by pests such as aphids, whiteflies, and mealybugs, or from exudates produced by the leaves of specific plants. These fungi are characterized by black, patchy colonies on plant surfaces (Jouraeva et al. 2006; Hongsanan et al. 2015). Species of Capnodiaceae dominate sooty mould communities, as their hyphal layers cover the plant surface, blocking sunlight and thereby interfering with leaf photosynthesis. In addition, the hyphal layers trap heat on the leaf surface. Overall, they not only reduce photosynthetic efficiency but also increase the plant’s own energy consumption, ultimately affecting plant growth and crop yields (Chomnunti et al. 2011; Abdollahzadeh et al. 2020).

Capnodiaceae fungi are widely distributed in tropical and subtropical regions (Chomnunti et al. 2011; Hongsanan et al. 2020). Nevertheless, the currently documented species diversity remains insufficient to support in-depth investigations of these fungi, particularly with respect to their ecological roles and interactions among fungi, insects, and host plants. China, despite its immense biodiversity, has so far reported only four species of Capnodiaceae, including three newly described species (*Conidiocarpus fici-septicae*, *Hyphocapnodia sichuanensis*, and *C. cinnamomeus*) and one newly recorded species (*C. caucasicus*) (Lu et al. 2022; Tennakoon et al. 2022; Thungdee et al. 2023; Tian et al. 2024; Yang et al. 2025). These taxa were described from different regions and host plants, including *C. fici-septicae* from fallen leaves of *Ficus septica* (Moraceae) (Tennakoon et al. 2022), *H. sichuanensis*, the sole species of the monotypic genus *Hyphocapnodia* from Sichuan Province (Lu et al. 2022), *C. cinnamomeus* from leaves of *Cinnamomum japonicum* (Yang et al. 2025), and *C. caucasicus* from withered leaves of *Cryptomeria japonica* in southwestern China (Tian et al. 2024). Collectively, these limited records indicate that Capnodiaceae remain markedly underrepresented in China and suggest that the diversity of sooty mould fungi in this region is still poorly explored.

Consequently, further systematic studies are required to reveal additional species diversity and to clarify unresolved taxonomic issues within Capnodiaceae. In this study, we describe novel species of Capnodiaceae associated with *Schefflera macrostachya* and *Dalbergia assamica* from China. These findings expand the known geographic and host ranges of the family and provide a foundation for future studies on the diversity and ecology of sooty mould fungi.

## Materials and methods

### Sample collection and morphological observations

Sooty mould-infected leaves were collected from *Schefflera macrostachya* in Guangdong Province and from *Dalbergia assamica* in Guizhou Province, China, in early spring (24 March 2025) and mid-summer (14 July 2025), respectively. The samples were placed in paper envelopes, transported to the laboratory, and stored at 4 °C. Macromorphological characteristics, including colony characteristics (size, shape, elevation, margin, texture, and color) and the distribution of conidiomata, were observed and documented using a stereomicroscope (Keyence VHX-7000 Digital Microscope, Japan). For microscopic analysis, samples were prepared on slides with sterile water and examined under a compound optical microscope (Zeiss Axioscope 5, Germany) equipped with an AxioCam 208 color camera and a phase-contrast optical system. The shape, pigmentation, and dimensions of hyphae, conidiomata, ostioles, the position of the swollen region of the conidiomata, and conidia were photographed and recorded. All images for illustration and dimensional measurements were processed using Adobe Photoshop (2023, 24.0.0.59). The holotype was deposited at the Herbarium of IFRD (International Fungal Research & Development Centre; Institute of Highland Forest Science, Chinese Academy of Forestry, Kunming, China). The ex-type living culture was deposited at the Culture Collection of the Herbarium of IFRD (IFRDCC). The newly described species were registered in Index Fungorum, and the corresponding accession numbers were obtained (Index Fungorum 2026).

### DNA extraction, PCR amplifications, and sequencing

DNA extraction was performed with fresh fungal mycelia cultured on potato dextrose agar (PDA) at 26 °C in darkness for 2 weeks using the BEIWO Fungal DNA Extraction Kit (Hangzhou Beiwo Medical Technology Co., Ltd., catalog No. BW-GD2416), following the manufacturer’s protocol. Genomic DNA of the host plant was extracted using the Plant Genomic DNA Extraction Kit from Beijing Solarbio Science & Technology Co., Ltd. For fungal gene amplification, four molecular markers were targeted: the internal transcribed spacer (ITS) region, including 5.8S rDNA, using primers ITS1/ITS4 (White et al. 1990); the large subunit ribosomal RNA gene (LSU), using primers LR0R/LR5 (Vilgalys and Hester 1990); the translation elongation factor 1-α gene (*tef*1-α), using primers EF1-983F/EF1-2218R (Reynolds and Gilbert 2005); and the RNA polymerase II second largest subunit gene (*rpb*2), using primers RPB2-5F/RPB2-7cR (Liu et al. 1999). For the host plant, the partial *rbc*L gene was amplified using the primer pair SI_Forward and SI_Reverse, which were developed by Kress et al. (2009). The PCR reaction mixture had a total volume of 20 μl, consisting of 17.5 μl GoldenStar T6 Super PCR Mix Ver. 2 (1.1×), 1 μl DNA template, 1 μl forward primer, and 1 μl reverse primer. PCR cycling parameters were optimized for each molecular marker, with the key annealing temperatures specified as follows: ITS and LSU were amplified with an annealing temperature of 52 °C (35 cycles); *tef*1-α at 55 °C (35 cycles); and *rpb*2 with a two-step annealing protocol (60 °C for 5 cycles, followed by 54 °C for 35 cycles). Other standard PCR conditions included initial denaturation, denaturation, extension, and final hold steps, as described in the original protocols (Vilgalys and Hester 1990; White et al. 1990; Reynolds and Gilbert 2005; Kress et al. 2009). Detailed procedures for the PCR assays used in the present study are listed in Table 1. PCR products were analyzed using 1% agarose gel, which was prepared and, after complete solidification, placed in an electrophoresis tank containing buffer. DNA samples were loaded into the wells, and electrophoresis was performed at 100 V for 30 min. The gel was visualized using a gel imaging system. PCR products were considered qualified if clear, bright bands were observed at the expected positions corresponding to the target gene fragments. Qualified PCR products were sent to Tsingke Biotechnology Co., Ltd. (Beijing) for sequencing. The resulting valid sequences were deposited in GenBank (Table 2).

**Table 1.** The PCR conditions and the primers used in this study.

Gene	Primers	Sequence (5’–3’)	PCR cycles	References
ITS	ITS1	TCCGTAGGTGAACCTGCGG	(95 °C: 30 s, 52 °C: 45 s, 72 °C: 60 s) × 35 cycles	White et al. (1990)
	ITS4	TCCTCCGCTTATTGATATGC		
LSU	LR0R	ACCCGCTGAACTTAAGC	(95 °C: 30 s, 52 °C: 45 s, 72 °C: 60 s) × 35 cycles	Vilgalys and Hester (1990)
	LR5	ATCCTGAGGGAAACTTC		
*tef*1-*α*	EF1-983F	GCYCCYGGHCAYCGTGAYTTYAT	(95 °C: 30 s, 55 °C: 45 s, 72 °C: 60 s) × 35 cycles	Reynolds and Gilbert (2005)
	EF1-2218R	ATGACACCRACRGCRACRGTYTG		
*rpb*2	RPB2-5F	GAYGAYMGWGATCAYTTYGG	(94 °C: 45 s, 60 °C: 45 s, 72 °C: 2 min) × 5 cycles; (94 °C: 45 s, 54 °C: 45 s, 72 °C: 2 min) × 5 cycles; (94 °C: 45 s, 54 °C: 45 s, 72 °C: 2 min) × 30 cycles	Liu et al. (1999)
	RPB2-7cR	CCCATRGCTTGYTTRCCCAT		
*rbc*L	SI_Forward	ATGTCACCACAAACAGAGACTAAAGC	(94 °C: 30 s, 55 °C: 30 s, 72 °C: 60 s) × 35 cycles	Kress et al. (2009)
	SI_Reverse	GTAAAATCAAGTCCACCRCG		

**Table 2.** Taxa used in the phylogenetic analysis of Capnodiaceae and their corresponding GenBank accession numbers.

Species	Strain	GenBank Accession Number				Reference
		ITS	LSU	*tef*1-*α*	*rpb*2	
*Capnodium aciculiforme*	CBS 892.73	-	-	GU349045	-	Schoch et al. (2009)
*Capnodium alfenasii* ^T^	CBS 146151	MN749233	MN749165	MN829346	MN829260	Abdollahzadeh et al. (2020)
*Capnodium blackwelliae* ^T^	CBS 133588	MN749235	-	-	-	Abdollahzadeh et al. (2020)
*Capnodium coartatum*	CPC 17779	MN749236	MN749167	MN829348	MN829262	Abdollahzadeh et al. (2020)
*Capnodium coartatum*	SDBR-CMU477	OR458575	OR458609	OR523660	-	Thungdee et al. (2023)
*Capnodium coffeae* ^T^	CBS 147.52	DQ491515	GU214400	DQ471089	KT216519	Spatafora et al. (2006)
*Capnodium coffeicola* ^ET^	MFLUCC 15-0206	KU358921	KU358920	-	-	Ariyawansa et al. (2015)
*Capnodium gamsii*	CBS 146153	MN749238	MN749168	MN829349	MN829263	Abdollahzadeh et al. (2020)
*Capnodium gardeniarum*	CPC 14327	-	GU301807	GU349054	GU371743	Schoch et al. (2009); Li et al. (2020)
*Capnodium neocoffeicola* ^T^	CBS 139614	MN749242	MN749172	MN829353	MN829267	Abdollahzadeh et al. (2020)
*Capnodium paracoartatum*	MFLUCC 14-0282	NR_169716	NG_073832	-	-	Li et al. (2020)
*Capnodium paracoffeicola* ^T^	CBS 139616	MN749244	MN749174	MN829355	MN829269	Abdollahzadeh et al. (2020)
*Chaetocapnodium insulare* ^T^	CBS 146159	MN749248	NG_068681	-	-	Abdollahzadeh et al. (2020)
*Chaetocapnodium magnum* ^T^	CBS 153154	PV583761	PV583758	PV591868	PV591871	Czachura et al. (2025)
*Chaetocapnodium polonicum* ^T^	CBS 153156	PV583763	PV583760	PV591870	PV591873	Czachura et al. (2025)
*Chaetocapnodium siamensis* ^T^	MFLUCC 13-0778	-	KP744479	-	-	Hongsanan et al. (2015)
*Chaetocapnodium tanzanicum* ^T^	CBS 145.79	MN749253	MN749182	MN829365	MN829280	Abdollahzadeh et al. (2020)
*Chaetocapnodium thailandense* ^T^	CBS 139619	MN749254	MN749183	MN829366	MN829281	Abdollahzadeh et al. (2020)
*Conidiocarpus caucasicus* ^T^	GUMH 937	-	KC833050	-	-	Bose et al. (2014)
*Conidiocarpus cinnamomi* ^T^	SICAU 23-0100	PP736390	PP732745	PP779591	PP782090	Yang et al. (2025)
*Conidiocarpus fici*-*septicae*^T^	MFLUCC 19-0072	MW063143	MW063206	-	-	Tennakoon et al. (2021)
*Conidiocarpus guilanensis* ^T^	IRAN 2474C	MG906804	-	-	-	Khodaparast et al. (2020)
*Conidiocarpus nanshanense* ^T^	IFRDCC 25-0001	PX668540	PX668542	PX682093	PX687868	This study
*Conidiocarpus siamensis* ^T^	MFLUCC 10-0061	KU358923	JN832607	-	-	Hongsanan et al. (2015)
*Heteroconium citharexyli* ^T^	CPC 13957	HM628776	HM628775	-	-	Cheewangkoon et al. (2012)
*Hyphocapnodia sichuanensis* ^T^	CGMCC 3.23573	ON603981	ON603986	ON646697	ON646698	Lu et al. (2022)
*Leptoxyphium cacuminum* ^T^	MFLUCC 10-0059	-	JN832603	-	-	Chomnunti et al. (2011)
*Leptoxyphium citri*	CBS 146162	MN749267	MN749195	MN829378	-	Abdollahzadeh et al. (2020)
*Leptoxyphium citri* ^T^	CBS 451.66	MN749266	KF902094	GU349039	GU371727	Abdollahzadeh et al. (2020)
*Leptoxyphium fumago*	CBS 123.26	MH854862	GU214430	GU349051	-	Schoch et al. (2009)
*Leptoxyphium glochidion* ^T^	IFRDCC 2651	KF982307	KF982308	-	-	Yang et al. (2014)
*Leptoxyphium kurandae*	CBS 129530	JF951150	JF951170	MN829379	MN829295	Crous et al. (2011)
*Leptoxyphium madagascariense* ^T^	CBS 124766	MH863407	MH874923	MN829380	MN829296	Abdollahzadeh et al. (2020)
*Phaeoxyphiella australiana* ^T^	CBS 146169	MN749292	MN749220	MN829406	MN829322	Abdollahzadeh et al. (2020)
*Phaeoxyphiella phylicae* ^T^	CBS 146170	MN749291	MN749219	MN829405	MN829321	Abdollahzadeh et al. (2020)
*Phragmocapnias betle*	CPC 17762	MN749293	MN749221	MN829407	MN829323	Abdollahzadeh et al. (2020)
*Phragmocapnias betle*	MFLUCC 10-0050	-	JN832605	-	-	Bose et al. (2014)
*Phragmocapnias betle* ^ET^	MFLUCC 10-0053	KU358922	JN832606	-	-	Bose et al. (2014)
*Phragmocapnias plumeriae* ^T^	MFLUCC 15-0205	KU358919	KU358918	-	-	Ariyawansa et al. (2015)
*Polychaeton cengongense* ^T^	IFRDCC 25-0002	PX668539	PX668541	PX682092	PX682094	This study
*Polychaeton citri*	CBS 116435	GU214649	GU214469	MN829394	MN829310	Crous et al. (2009)
*Scolecoxyphium blechni* ^T^	CBS 146174	MN749296	MN749224	MN829412	MN829328	Abdollahzadeh et al. (2020)
*Scolecoxyphium blechnicola* ^T^	CBS 146175	MN749297	MN749225	MN829413	MN829329	Abdollahzadeh et al. (2020)
*Scolecoxyphium leucadendri* ^T^	CBS 146176	MN749298	MN749226	MN829414	MN829330	Abdollahzadeh et al. (2020)
*Scolecoxyphium phylicae* ^T^	CBS 146177	MN749299	MN749227	MN829415	MN829331	Abdollahzadeh et al. (2020)

Sequences and details of strains obtained in this study are shown in bold. Abbreviations: T and ET indicate type species. - indicates unavailable data or sequence.

### Phylogenetic analyses

Gene sequencing results obtained from the biotechnology company were analyzed by examining DNA sequence peaks using BioEdit version 7.2.5 (Hall 1999). Phylogenetic analyses were performed using a one-stop software for fungal phylogeny (OFPT) (Zeng et al. 2023), which first requires preparation of reference sequence data from the NCBI database (https://www.ncbi.nlm.nih.gov/) and newly collected sequences. The specific workflow was as follows: gene sequences were downloaded from NCBI. Afterwards, datasets for each gene region were aligned independently using MAFFT (https://mafft.cbrc.jp/alignment/server/) (Katoh and Standley 2013). These alignments were then trimmed using TrimAl with the “gappyout” method (Capella-Gutiérrez et al. 2009). Phylogenetic trees were constructed using the Bayesian inference (BI) method (Ronquist et al. 2012), and bootstrap (BS) values were obtained. Aligned files (NEX format) were run in MrBayes v. 3.2.6, and the selection of the optimal nucleotide substitution model was performed using MrModeltest v. 2.3. Bayesian inference was conducted using a Markov chain Monte Carlo (MCMC) approach with two independent runs of four Markov chains each. The analysis was run for 50 million generations, sampling every 100 generations, and was terminated when the average standard deviation of split frequencies between runs dropped below 0.01. In total, 1,000,000 trees were generated, of which the first 25% were discarded as burn-in prior to constructing the majority-rule consensus tree. Posterior probabilities (PP) were calculated for each clade. The resulting tree files were visualized using FigTree v. 1.4.3, and the phylogenetic trees were further edited and formatted in Adobe Illustrator CC 2024 (AI) before being saved as PDF files.

## Results

### Phylogenetic analyses

The alignment included 45 strains and contained a total of 3223 characters, including gaps, with 1–445, 446–1,280, 1,281–2,332, and 2,333–3,223 corresponding to ITS, LSU, *rpb*2, and *tef*1-α, respectively. The optimal nucleotide substitution models were TIM2e+G4, TNe+R2, TIM2e+I+G4, and TIM3+F+I+G4, respectively. The phylogenetic analysis of the concatenated dataset produced a best-scoring tree with a final maximum likelihood optimization value of –16613.553.

**Figure 1.**
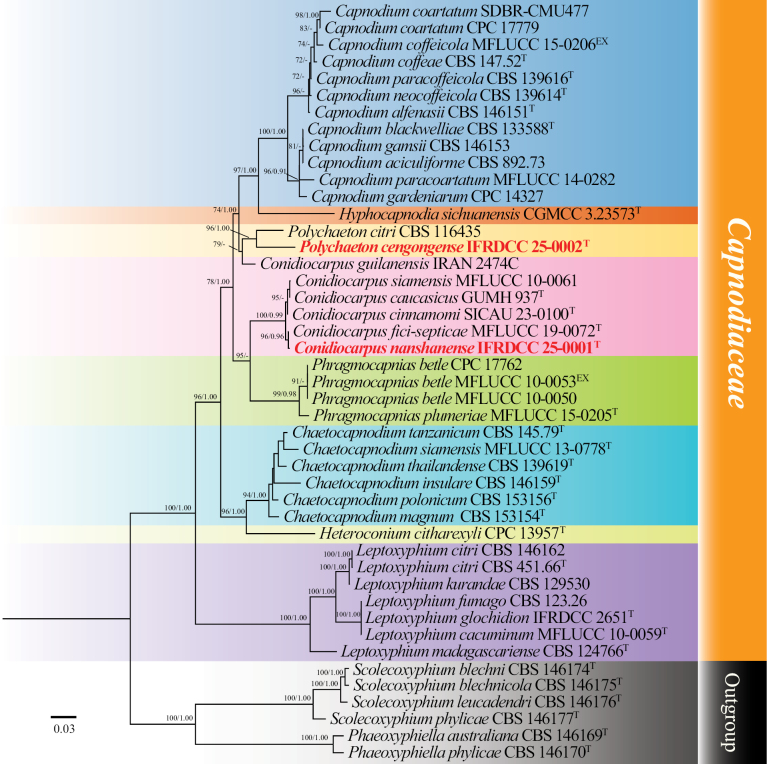
Phylogenetic tree generated from ML analysis based on the concatenated datasets of LSU, ITS, *tef*1-α, and *rpb*2 gene regions. All type strains are indicated by a superscript T, and the new isolate is highlighted in bold red. The phylogenetic tree was rooted using Readerielliopsidaceae taxa (Abdollahzadeh et al. 2020) as the outgroup. Bootstrap support (BS) values that are equal to or exceed 70% and Bayesian posterior probabilities (PP) values that are equal to or exceed 0.9 are shown above the nodes. Type strains: superscript T and ET; new isolate: red bold; unavailable sequences are denoted by -.

The tree topology from BI was similar to that from ML. Thus, only the ML tree is shown. Capnodiaceae comprises eight genera: *Conidiocarpus*, *Phragmocapnias*, *Capnodium*, *Hyphocapnodia*, *Polychaeton*, *Chaetocapnodium*, *Heteroconium*, and *Leptoxyphium*. The genera *Conidiocarpus* (6 representative sequences), *Phragmocapnias* (4), *Capnodium* (12), *Hyphocapnodia* (1), *Polychaeton* (2), *Chaetocapnodium* (6), *Heteroconium* (1), and *Leptoxyphium* (8) form a monophyletic group. In this study, the two new species belong to different genera, with *Conidiocarpus* sister to *Phragmocapnias* and *Polychaeton* sister to *Capnodium* (Fig. 1).

### Taxonomy

#### Conidiocarpus

Taxon classificationFungiCapnodialesCapnodiaceae

Woron., Ann. Mycol. 24: 250 (1927)

9DAAF616-CB53-5D99-A52E-8B5CD7F2D17E

##### Type species.

*Conidiocarpus caucasicus* Woron., Key to fungi (fungi imperfecti) 2: 743 (1917).

##### Notes.

*Conidiocarpus* was recognized as the asexual morph of *Phragmocapnias* (Hughes 1976) and was treated as a synonym of *Phragmocapnias* (Hughes 1976; Chomnunti et al. 2011; Chomnunti et al. 2014; Hongsanan et al. 2015). Following the nomenclatural priority principle, Bose et al. (2014) adopted Hughes’ (1976) viewpoint and transferred species from *Phragmocapnias* to *Conidiocarpus*. However, Abdollahzadeh et al. (2020) proposed that these two are separate, independent genera based on morphological and phylogenetic analyses. According to MycoBank (2026) (https://www.mycobank.org, January 2026), the genus *Conidiocarpus* includes fifteen validly recognized species, including *C. asiaticus*, *C. betel*, *C. callitris*, *C. caucasicus*, *C. fici-septicae*, *C. fuliginodes*, *C. guilanensis*, *C. heliconiae*, *C. imperspicuus*, *C. longicollus*, *C. penzigii*, *C. philippinensis*, *C. plumeriae*, *C. siamensis*, and *C. cinnamomi* (Abdollahzadeh et al. 2020; Khodaparast et al. 2020; Tennakoon et al. 2022; Pem et al. 2024; Yang et al. 2024; Yang et al. 2025). *Conidiocarpus* is distinguished from other genera by the following characteristics: the swollen part of the sporogenous structure is located in the middle–upper part of the conidioma, accompanied by a long stipe and an elongated base (Chomnunti et al. 2011).

#### Conidiocarpus nanshanense

Taxon classificationFungiCapnodialesCapnodiaceae

J.P. Sun & X.Y. Zeng sp. nov.

75EFC5BD-8AAC-5C06-9307-FBA7794F4006

Index Fungorum: IF904721

Fig. 2

##### Etymology.

Refers to the type locality, “Nanshan Park.”

**Figure 2.**
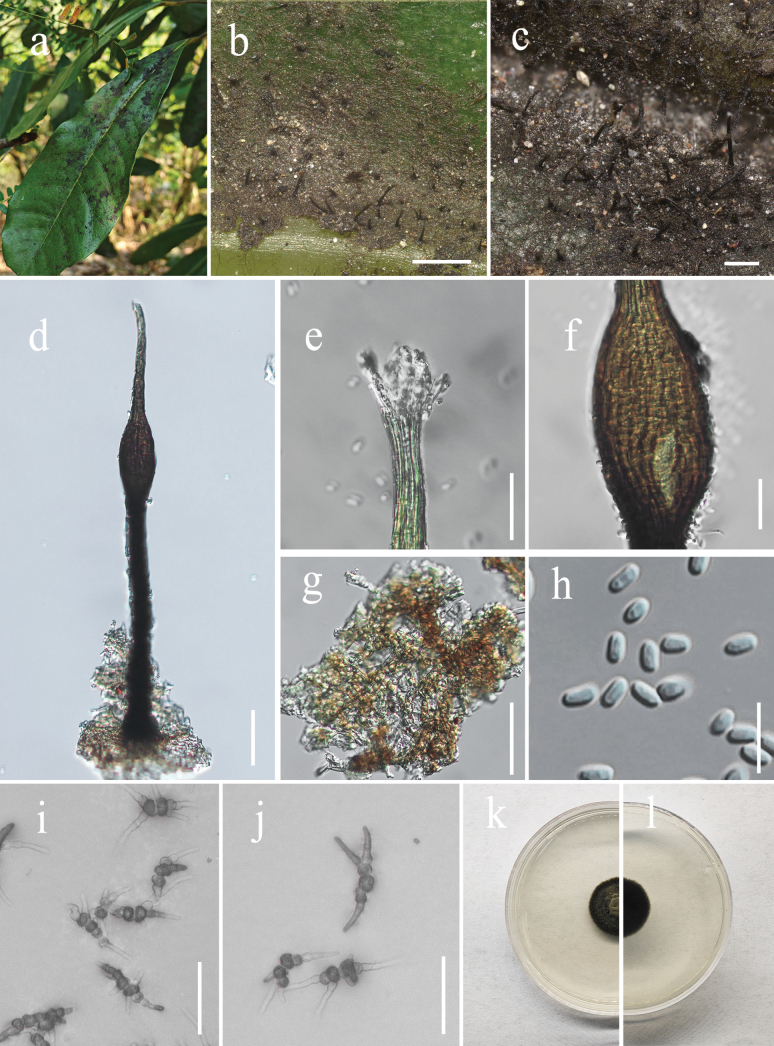
*Conidiocarpus nanshanense* (IFRD 99050). **a**. Black mycelium covering the leaf surface; **b, c**. Conidiomata on the host; **d**. Conidioma; **e**. Ostiole neck; **f**. A prominently ovoid-swollen conidiogenous region; **g**. Mycelial network; **h**. Conidia; **i, j**. Germinated conidia; **k**. Front view of the colony on the PDA; **l**. Reverse view of the colony on the PDA. Scale bars: 500 µm (**b**); 50 µm (**c, d, g, i, j**); 20 µm (**e, f**); 10 µm (**h**).

##### Holotype.

IFRD 99050.

##### Description.

***Epiphytic*** on the leaf surface of *Schefflera macrostachya*, forming a sooty coating on adaxial surface (Fig. 2). ***Thallus*** composed of brown, septate, ellipsoidal, smooth-walled hyphae. ***Asexual morph*. *Conidiomata*** (342–493 (–513) × 27–53 µm, x̄ = 423 × 39 µm, n = 15) long, pycnidial, elongate, superficial, stipitate, with a long stalk (118–280 µm, x̄ = 226 µm, n = 15), black, rigid, a distinct neck (66–120 µm, x̄ = 101 µm, n = 15) height, and a prominently ovoid-swollen conidiogenous region, the swollen area producing conidia inside. ***Conidiomata apex*** gradually light brown to hyaline, with a circular ***ostiole*** (7–14 (–17) µm, x̄ = 11 µm, n = 15) diam, consisting of rectangular, compact cells, surrounded by hyaline hyphae. ***Conidiogenous region***, located in the upper-middle part of the conidiomata, (25–47 µm, x̄ = 39 µm, n = 15) wide, brown, composed of cylindrical, thin-walled cells. ***Conidia*** 4.1–6.4 × 2.4–3.3 µm (x̄ = 5 × 2.7 µm, n = 30), hyaline, single-celled, ellipsoidal, smooth-walled, guttulate with 1–2 distinct refractive oil droplets, exuding in creamy masses from the ostiole. ***Sexual morph***. Undetermined.

##### Culture characteristics.

Colonies growing on PDA reaching 16 mm in diam. after 14 days at 26 °C in the dark, colony surface gradually erumpent, with hyphae growing downward and immersed in the medium, olivaceous. Aerial hyphae pale to dark brown, cylindrical, distinctly verrucose, branched, with thin, inconspicuous septa (not constricted), walls becoming thickened.

##### Material examined.

China • Guangdong Province, Shenzhen City, Nanshan Park, on living leaves of *Schefflera macrostachya*. (22°50'25"N, 113°91'26"E), 24 March 2025, Jipeng Sun, (IFRD 99050, holotype), ex-type living culture IFRDCC 25-0001.

##### Notes.

In the phylogenetic tree, *C. nanshanense* (IFRDCC 25-0001) clusters with *C. fici-septicae* (MFLUCC 19-0072), with BS/PP support values of 96%/0.96, and forms a sister clade with the strains *C. cinnamomi* (SICAU 23-0100), *C. siamensis* (MFLUCC 10-0061), and *C. caucasicus* (GUMH 937). There are 99.25% (535/539, 4 gaps) and 99.77% (879/881, 1 gap) similarities in the ITS and LSU regions between *C. nanshanense* (IFRDCC 25-0001) and *C. fici-septicae* (MFLUCC 19-0072), respectively. Comparison of *tef*1-α and *rpb*2 sequences could not be conducted due to the lack of sequence data for *C. fici-septicae* (MFLUCC 19-0072). Tian et al. (2024) reported the first Chinese record of *C. caucasicus* (UESTCC 23.0246) and supplemented the molecular data for the ex-type strain *C. caucasicus* (GUMH 937) with ITS, LSU, *tef*1-α, and *rpb*2 sequences. Multi-gene alignment between *C. nanshanense* (IFRDCC 25-0001) and *C. caucasicus* (UESTCC 23.0246) revealed the following sequence similarities: ITS 99.80% (511/512, 0 gaps), LSU 99.63% (806/809, 0 gaps), *tef*1-α 97.90% (841/859, 0 gaps), and *rpb*2 99.29% (981/988, 0 gaps). Multi-gene alignment between *C. nanshanense* (IFRDCC 25-0001) and *C. cinnamomi* (SICAU 23-0100) showed the sequence similarities as follows: ITS 98.88% (528/534, 2 gaps) and LSU 99.10% (877/885, 1 gap). In terms of morphological characteristics, *C. nanshanense* (IFRDCC 25-0001) differs from *C. fici-septicae* (MFLUCC 19-0072) by possessing larger conidiomata (342–493 µm vs. 240–280 μm), a broader base (27–53 μm vs. 7–8 μm), a wider ostiole (7–17 μm vs. 6–9 μm), and larger conidia (5 × 2.7 vs. 4.5 × 1.5 μm) (Tennakoon et al. 2021). Compared to *C. caucasicus* (GUMH 937), *C. nanshanense* (IFRDCC 25-0001) has smaller conidiomata (342–493 µm vs. 420–930 μm), narrower ostioles (7–14 μm vs. 14–28 μm), and larger conidia (4.1–6.4 × 2.4–3.3 µm vs. 3.5–5 × 1.5–2 μm) (Khodaparast 2006). When compared with *C. cinnamomi* (SICAU 23-0100), *C. nanshanense* (IFRDCC 25-0001) exhibits smaller conidiomata (342–493 µm vs. 500–860 μm) and wider ostioles (7–17 μm vs. 4–8 μm) (Yang et al. 2025). However, a morphological comparison with *C. siamensis* (MFLUCC 10-0061), which was isolated from an unknown host, could not be conducted due to the lack of a detailed morphological description for this strain (Chomnunti et al. 2011; Abdollahzadeh et al. 2020). *Conidiocarpus caucasicus*, *C. fici-septicae*, and *C. cinnamomi* were isolated from *Citrus* sp. (Rutaceae, Sapindales), *Ficus septica* (Moraceae, Rosales), and *Cinnamomum japonicum* (Lauraceae, Laurales), respectively (Khodaparast 2006; Tennakoon et al. 2021; Yang et al. 2025), whereas *C. nanshanense* was discovered on *Schefflera macrostachya* (Araliaceae, Apiales) in this study. Accordingly, the species represented by this strain is described as a new species, *C. nanshanense*.

#### Polychaeton

Taxon classificationFungiCapnodialesCapnodiaceae

(Pers.) Lév., Dictionnaire universelle d’histoire naturelle 8: 493 (1846)

9F113CA0-D06F-5E4F-9DCC-22A525B1FFD7

##### Type species.

*Polychaeton quercinum* (Pers.) Kuntze, Revisio generum plantarum 3 (3) (1891).

##### Notes.

*Polychaeton* was originally established by Persoon (1822) as a subgenus within *Fumago* and was later elevated to generic rank by Léveillé (1847). The taxonomic concept of *Polychaeton* has subsequently been re-evaluated by Hughes (1976) and Chomnunti et al. (2011). Hughes (1976) recognized *Polychaeton citri* and *P. quercinum* as representative species of the genus and formally designated *P. quercinum* as the lectotype. In contrast, Chomnunti et al. (2011) interpreted *Capnodium* as the sexual morph associated with *Polychaeton* and, in accordance with the “one fungus = one name” principle, adopted *Capnodium* as the accepted genus. According to MycoBank (2026) (https://www.mycobank.org, January 2026), the genus *Polychaeton* currently comprises 19 species, although molecular sequence data are available for only *Polychaeton citri*. *Polychaeton* is distinguished from other genera by the position of the swollen conidiogenous region in the middle to lower part of the conidiomata and by the absence of a stalk (Chomnunti et al. 2011).

#### Polychaeton cengongense

Taxon classificationFungiCapnodialesCapnodiaceae

J. P. Sun & X. Y. Zeng sp. nov.

C61BC308-66F8-5CC6-8575-F5BA2970D273

Index Fungorum: IF904722

Fig. 3

##### Etymology.

Refers to the type locality, “Cengong County.”

**Figure 3.**
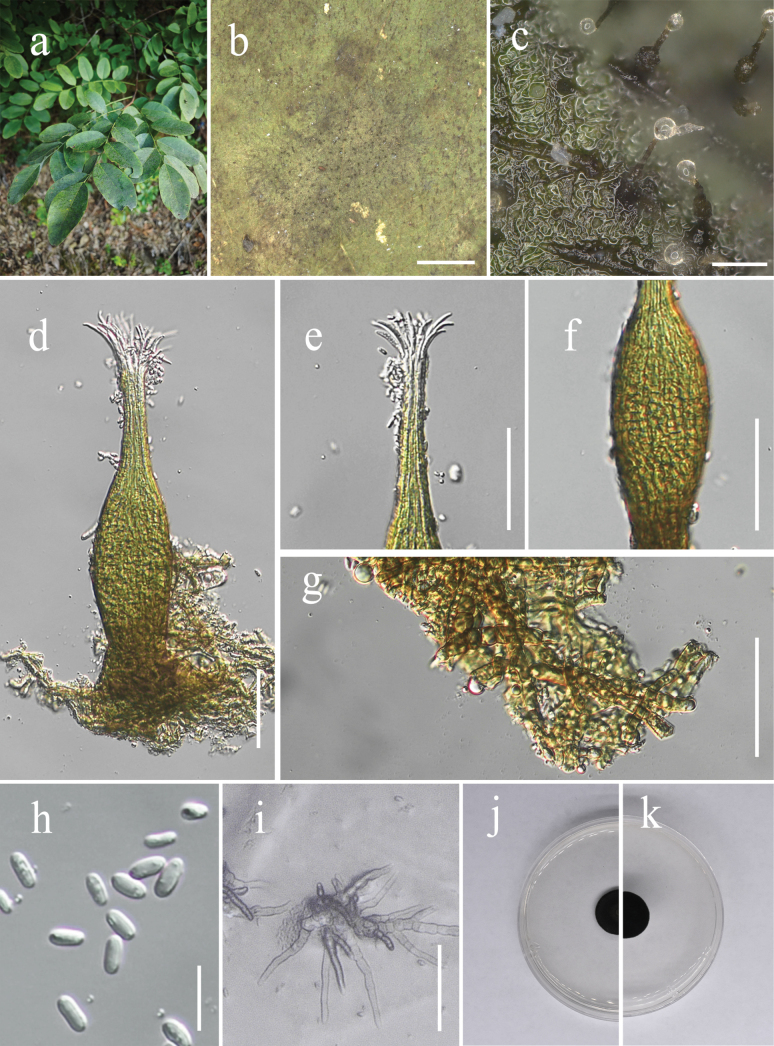
*Polychaeton cengongense* (IFRD 99051). **a**. Black mycelium covering the leaf surface; **b, c**. Conidiomata on the host; **d**. Conidioma; **e**. Ostiole neck; **f**. A prominently ovoid-swollen conidiogenous region; **g**. Mycelial network; **h**. Conidia; **i**. Germinated conidia; **j**. Front view of the colony on the PDA; **k**. Reverse view of the colony on the PDA. Scale bars: 1000 µm (**b**); 100 µm (**c**); 50 µm (**d–g**); 10 µm (**h**); 25 µm (**i**).

##### Holotype.

IFRD 99051.

##### Description.

***Epiphytic*** on the leaf surface of *Dalbergia assamica*, forming a sooty coating on adaxial surface (Fig. 3). ***Thallus*** composed of brown, septate, ellipsoidal, smooth-walled hyphae. Asexual morph**. *Conidiomata*** (182–263 × 19–46 (–54) µm, x̄ = 224 × 34 µm, n = 20) long, pycnidial, elongate, superficial, stipitate, lack stalk, with a distinct neck (62–125 µm, x̄ = 95 µm, n = 20) height, a prominently ovoid-swollen conidiogenous region, the swollen area producing conidia inside. ***Conidiomata apex*** light brown to hyaline, with a circular ***ostiole*** (10–23 µm, x̄ = 16 µm, n = 20) diam, consisting of rectangular, compact cells, surrounded by hyaline hyphae. ***Conidiogenous region***, located in the lower-middle part of the conidiomata, (30–62 µm, x̄ = 37 µm, n = 20) wide, brown, composed of cylindrical, thin-walled cells. ***Conidia*** 4.2–6.5 × 1.7–4 µm (x̄ = 5.1 × 2.3 µm, n = 30), hyaline, single-celled, ellipsoidal, smooth-walled, guttulate with 1–2 distinct refractive oil droplets, exuding in creamy masses from the ostiole. ***Sexual morph***. Undetermined.

##### Culture characteristics.

Colony up to 15.6 mm in diam on PDA at 26 °C after 14 days in darkness. Colony superficial, velvety, with an entire margin, olivaceous-green. Aerial hyphae pale to dark brown, cylindrical, distinctly verrucose (with 1–2 μm tall warts), branched, with thin, inconspicuous septa (not constricted), walls becoming thickened.

##### Material examined.

China • Guizhou Province, Cengong County, on living leaves of *Dalbergia assamica* (27°44'20"N, 108°44'20"E), 14 July 2025, Jipeng Sun, (IFRD 99051, holotype), ex-type living culture IFRDCC 25-0002.

##### Notes.

In the phylogenetic tree, *Polychaeton cengongense* (IFRDCC 25-0002) forms a sister clade to *Polychaeton citri* (CBS 116435), with BS/PP support values of 96%/1, respectively. *Polychaeton cengongense* (IFRDCC 25-0002) and *P. citri* (CBS 116435) have 99.06% (508/518, 3 gaps) and 93.91% (756/805, 0 gaps) similarity in the ITS and LSU regions, respectively, while comparison of their *tef*1-α and *rpb*2 regions reveals 93.91% (756/805, 0 gaps) and 89.67% (738/823, 0 gaps) sequence similarity, respectively. In morphological data, compared to *P. citri* (CBS 116435), *P. cengongense* (IFRDCC 25-0002) has smaller conidiomata (182–263 μm vs. 345–391 μm), narrower bases (19–46 μm vs. 36–40 μm), wider ostioles (10–23 μm vs. 13–15 μm), and smaller conidia (5.1 × 2.3 μm vs. 6.5 × 5 μm) (Chomnunti et al. 2011). *P. citri* was isolated from *Citrus aurantium* (Rutaceae, Sapindales), while *P. cengongense* in this study was obtained from *Dalbergia assamica* (Fabaceae, Fabales), representing a new host record for this species. The hosts of these two species exhibit remarkable differences at the higher taxonomic ranks of family and order with no overlap at all. Therefore, the species represented by this strain is described as a new species, *P. cengongense*.

## Discussion

China, one of the world’s most significant biodiversity hotspots, harbors a remarkably rich and underexplored diversity of sooty mould fungi (Capnodiaceae). Although the family is globally distributed across tropical and subtropical ecosystems, only a few Capnodiaceae species have been formally documented in China (Lu et al. 2022; Tennakoon et al. 2022; Thungdee et al. 2023; Tian et al. 2024; Yang et al. 2025), leaving a significant gap in our understanding of their ecological roles and evolutionary history. The discovery of *Conidiocarpus nanshanense* and *Polychaeton cengongense* highlights the vast, untapped fungal diversity yet to be uncovered within China’s subtropical forests, especially in undersampled areas such as Guizhou and Guangdong Provinces. These findings not only expand the species inventory of Capnodiales in China but also highlight the urgent need for more systematic surveys to better understand the ecological interactions and biogeographic patterns of sooty mould fungi in this megadiverse country. Such efforts are crucial for establishing a solid taxonomic framework and guiding conservation strategies for China’s unique fungal flora.

Our morphological and phylogenetic analyses of the isolated strains confirm the distinctiveness of *Conidiocarpus nanshanense* and *Polychaeton cengongense* as new species. Species delimitation was supported by an integrative taxonomic approach combining detailed morphological characterization and multi-gene phylogenetic analysis. Both new species form well-supported clades in the phylogenetic trees and exhibit consistent morphological and molecular differences from closely related taxa. These findings enrich the known species diversity of *Conidiocarpus* and *Polychaeton* in China and provide crucial morphological and molecular data to facilitate further taxonomic studies of Capnodiales.

In traditional understanding, sooty mould fungi are regarded as “harmful fungi” because they cover leaf surfaces and interfere with photosynthesis (Chomnunti et al. 2011; Thungdee et al. 2023). However, field observations in this study revealed that the colonization densities of *Conidiocarpus nanshanense* and *Polychaeton cengongense* show a positive correlation with the aphid population density on host plants, without causing host defoliation. This suggests that sooty mould fungi may be obligate decomposers of insect honeydew. By decomposing honeydew secreted by aphids, they prevent secondary fungal infections on leaves induced by honeydew accumulation and thus actually act as “buffers” in plant–insect interactions. Experimental evidence has confirmed that certain species of sooty mould fungi can produce secondary metabolites, including those from three genera, *Capnodium*, *Leptoxyphium*, and *Trichomerium* (Herath et al. 2012; Haituk et al. 2022). These secondary metabolites inhibit the growth of common airborne fungi such as *Penicillium* and *Mucor*. Currently, the taxonomic status of species in the genus *Conidiocarpus* remains unclear. For some closely related species, such as *C. fici-septicae*, only ITS/LSU sequences are available, lacking markers with higher resolution (*tef*1-α and *rpb*2), which results in inaccurate species delimitation. To address the issue of insufficient molecular markers, priority should be given to supplementing *tef*1-α, *rpb*2, and other gene sequences for type strains of sooty mould fungi, thereby establishing a multi-gene reference database.

The morphological characteristics of sooty mould fungi (such as conidiomata and conidia dimensions) are prone to morphological convergence across taxa under environmental influences, while the same species may also exhibit morphological divergence due to varying environmental conditions (Abdollahzadeh et al. 2020; Thungdee et al. 2023; Tian et al. 2024). This not only leads to the misidentification of distinct taxa as the same species and to the erroneous division of a single species into multiple taxa, but also results in ambiguity in the taxonomic boundaries within *Conidiocarpus*. However, *C. nanshanense* and *C. fici-septicae* exhibit high morphological similarity; combined phylogenetic inference based on the multi-locus dataset (ITS+LSU+*tef*1-α+*rpb*2) reveals that they are not conspecific taxa. Instead, they cluster within the same clade of the genus *Conidiocarpus* as distinct divergent lineages, and this phylogenetic relationship is supported by strong statistical evidence (BS/PP = 96%/0.96). This finding suggests that morphological trait convergence does not imply complete congruence in phylogenetic relationships. This result supports the hypothesis that the genus *Conidiocarpus* is a monophyletic group (Wheeler et al. 2013; Williams et al. 2016) and revises the traditional morphological classification criterion that delimits *Conidiocarpus* species based on conidiomata size. In this study, the conidiomatal sizes and swollen regions of *C. nanshanense* and *C. caucasicus* overlap, whereas the ostiole width and spore length–width ratio differ. Prior to this study, most known species of the genus *Conidiocarpus* have been reported from Southeast Asia, Thailand, and West Asia, Iran (Chomnunti et al. 2011; Khodaparast et al. 2020). The discovery of the new *Conidiocarpus* species in China reveals the characteristics of both connection and isolation between the sooty mould fungal flora of Southwest China and that of Southeast Asia.

In the taxonomic studies of sooty mould fungi, Chomnunti et al. (2011) first clarified the taxonomic correspondence between *Polychaeton* and *Capnodium* by constructing a phylogenetic tree based on multiple specimens of polychaeton-like taxa and combining SSU and LSU gene sequences. For their part, Abdollahzadeh et al. (2020) retrieved *Polychaeton citri* CBS 116435 from the Westerdijk Fungal Biodiversity Institute (CBS), Utrecht, the Netherlands. This strain was isolated from the host plant *Citrus aurantium* in Iran (Chomnunti et al. 2011; Hyde et al. 2011). Through rejuvenation culture on oatmeal agar (OA) and synthetic low nutrient agar (SNA), they found that the morphology of its conidiomata exhibited medium-dependent differences: on SNA medium, the strain possessed swollen conidiogenous structures and a slender neck with a short stalk, whereas on OA medium, its neck was further elongated, while the swelling characteristic was absent. By contrast, the conidiomata of taxa in the genus *Conidiocarpus* maintained a stable morphology of a swollen main body, long neck, and long stalk on both media. Despite the studies mentioned above confirming the taxonomic independence of *Polychaeton*, numerous controversies remain regarding the accurate delimitation of intra-generic species. Meanwhile, the new species *Polychaeton cengongense* described in this study represents the first record of *Polychaeton* on the host *Dalbergia assamica* in Guizhou Province, China, and adds to the literature, laying a critical foundation for clarifying the taxonomic boundaries of this genus and improving species identification.

The successful amplification of the *rbc*L gene from *Polychaeton cengongense* provides molecular confirmation of its host association with *Dalbergia assamica*. The complete *rbc*L gene sequence of the host, with a BLAST 100% (573/573, 0 gaps) similarity, is provided in Suppl. material 1. Combined with previous reports of *Polychaeton* species on *Citrus aurantium* (Rutaceae, Sapindales) (Chomnunti et al. 2011), this indicates ecological differentiation in host associations of this genus within woody plant taxa. For *Conidiocarpus nanshanense*, the inability to obtain host *rbc*L data underscores the challenge of working with preserved, field-collected samples, where prolonged storage can lead to DNA degradation. However, the morphological confirmation of *Schefflera macrostachya* as its host, together with the distinct geographical distribution (Guizhou/Guangdong Province vs. Taiwan/Sichuan Province for closely related species), further supports the ecological differentiation of *C. nanshanense* and its status as a novel species. These host associations also contribute to our understanding of the diversity and host specificity of sooty mould fungi in subtropical China.

The two new species described in this study are not only new records of sooty mould fungal diversity in China but also provide novel scientific perspectives for research on Capnodiaceae along two dimensions: taxonomic revision and re-evaluation of ecological functions. Future research could focus on the phylogeography of global sooty mould fungi and their contributions to honeydew nutrient cycling to deepen the understanding of their taxonomy, evolution, and ecological functions.

## Supplementary Material

XML Treatment for Conidiocarpus

XML Treatment for Conidiocarpus nanshanense

XML Treatment for Polychaeton

XML Treatment for Polychaeton cengongense

## Additional information

### Use of AI

No use of AI was reported.

### Author ORCIDs

Jipeng Sun https://orcid.org/0009-0004-0065-6920

Entaj Tarafder https://orcid.org/0000-0002-3680-3433

Tao Chen https://orcid.org/0009-0004-7806-4503

Sinang Hongsanan https://orcid.org/0000-0003-0550-3152

Shengxi Chen https://orcid.org/0009-0005-4945-5094

Chada Norphanphoun https://orcid.org/0000-0002-5756-7206

Samantha C. Karunarathna https://orcid.org/0000-0001-7080-0781

Xiangyu Zeng https://orcid.org/0000-0003-1341-1004

### Data availability

All of the data that support the findings of this study are available in the main text or Supplementary Information.

## Supplementary materials

Supplementary material 1 *Dalbergia assamica* rbcL complete sequence Data type fasta https://binary.pensoft.net/file/1544461This dataset is made available under the Open Database License (http://opendatacommons.org/licenses/odbl/1.0/). The Open Database License (ODbL) is a license agreement intended to allow users to freely share, modify, and use this Dataset while maintaining this same freedom for others, provided that the original source and author(s) are credited. Jipeng Sun, Entaj Tarafder, Tao Chen, Sinang Hongsanan, Shengxi Chen, Chada Norphanphoun, Samantha C. Karunarathna, Xiangyu Zeng
